# Smoking intensity and urinary nicotine metabolites by socioeconomic status in the Heinz Nixdorf Recall study

**DOI:** 10.1186/s12889-022-12609-y

**Published:** 2022-02-14

**Authors:** Jan Hovanec, Tobias Weiß, Holger M. Koch, Beate Pesch, Thomas Behrens, Benjamin Kendzia, Marina Arendt, Nico Dragano, Susanne Moebus, Börge Schmidt, Thomas Brüning, Karl-Heinz Jöckel

**Affiliations:** 1 Institute for Prevention and Occupational Medicine of the German Social Accident Insurance, Institute of the Ruhr University Bochum (IPA), Bürkle-de-la-Camp-Platz 1, 44789 Bochum, Germany; 2 Institute for Medical Informatics, Biometry and Epidemiology, Essen University Hospital, Essen, Germany; 3 Department of Computer Science, University of Applied Sciences and Arts, Dortmund, Germany; 4 Institute of Medical Sociology, Medical Faculty, University of Düsseldorf, Düsseldorf, Germany; 5 Institute for Urban Public Health, Essen University Hospital, University Duisburg-Essen, Essen, Germany

**Keywords:** Information bias, Cotinine, Trans-3′-hydroxy-cotinine, Occupational status, Cigarettes per day

## Abstract

**Background:**

Smoking intensity, which is generally based on self-reported average cigarettes per day (CPD), is a major behavioural risk factor and strongly related to socioeconomic status (SES). To assess the validity of the CPD measure, correlations with objective markers of tobacco smoke exposure – such as urinary nicotine metabolites – were examined. Yet, it remains unclear, whether this correlation is affected by SES, which may indicate imprecise or biased self-reports of smoking intensity.

**Methods:**

We investigated the role of SES in the association between CPD and nicotine metabolites in current smokers among the participants of the population-based, prospective Heinz Nixdorf Recall Study. We determined urinary cotinine and additionally trans-3′-hydroxy-cotinine. SES was assessed by the International Socio-Economic Index of occupational status, and education. We calculated correlations (Pearson’s r) between logarithmised CPD and cotinine in subgroups of SES and analysed SES and further predictors of cotinine in multiple linear regression models separately by gender.

**Results:**

Median reported smoking intensity was 20 CPD in male and 19 CPD in female smokers. Men showed higher cotinine concentrations (median 3652 μg/L, interquartile range (IQR) 2279–5422 μg/L) than women (3127 μg/L, IQR 1692–4920 μg/L). Logarithmised CPD correlated moderately with cotinine in both, men and women (Pearson’s r 0.4), but correlations were weaker in smokers with lower SES: Pearson’s r for low, intermediate, and high occupational SES was 0.35, 0.39, and 0.48 in men, and 0.28, 0.43, and 0.47 in women, respectively. Logarithmised CPD and urinary creatinine were main predictors of cotinine in multiple regression models, whereas SES showed a weak negative association in women. Results were similar for trans-3′-hydroxy-cotinine.

**Conclusions:**

Decreasing precision of self-reported CPD was indicated for low SES in men and women. We found no strong evidence for biased self-reports of smoking intensity by SES.

**Supplementary Information:**

The online version contains supplementary material available at 10.1186/s12889-022-12609-y.

## Background

Elevated risks for mortality and many chronic diseases in lower socioeconomic status (SES) groups are partly attributable to higher smoking rates among these groups [1, 2]. Thus, valid information on smoking behaviour is needed to estimate smoking related risks particularly in studies of SES and health. Along with smoking status, duration and intensity are important components of smoking behaviour [3]. Most empirical studies use smoking information based on self-reports, and average smoking intensity is frequently retrospectively solicited as cigarettes per day (CPD), which is also used to calculate cumulative lifetime tobacco exposure.

Cotinine is the major metabolite of nicotine and further metabolised mainly to trans-3′-hydroxy-cotinine, both quantifiable in body fluids and frequently applied biomarkers for recent tobacco exposure [4]. Therefore, cotinine can be used to validate the current self-reported smoking status as well as the recent dose of tobacco exposure. However, CPD and cotinine or trans-3′-hydroxy-cotinine were correlated weakly and showed a non-linear relationship with a suggested ceiling effect at higher CPD consumption [5–8]. Possible explanations for a plateau of cotinine and other tobacco smoke constituents are biological saturation, different inhalation behaviour, and information bias in self-reported CPD [9]. Differences by SES would point to a possible reporting bias or differential inhalation habits, rather than biological causes. However, only few studies addressed the role of SES when validating smoking intensity by cotinine concentration, and results differed by indicators of SES [10, 11]. Therefore, we extended a previous analysis on urinary cotinine, smoking status and SES in a German population-based cohort [12]. We first analysed the association of CPD and cotinine in current smokers, and then investigated the influence of SES and other predictors of cotinine including urinary creatinine and age. Based on the general results for the CPD-cotinine association, our specific aim was to identify differences of self-reported CPD by SES, which may indicate imprecise or biased self-reports of smoking intensity.

## Methods

### Study population

The Heinz-Nixdorf Recall Study (HNR) is a population-based prospective cohort study with participants randomly selected from three neighbouring cities of the Ruhr area in Germany [13]. Here, we analysed self-reported current smokers using interview data and archived urine samples of the baseline examination conducted in 2000–2003. Additional information on occupational histories was gathered in the second follow-up of HNR in 2011–2014. The ethics committee of the Medical Faculty of the University Duisburg-Essen approved the study and all participants provided written informed consent.

The baseline study population of HNR consisted of 4814 participants. We subsequently excluded participants with missing information on smoking habits (*n* = 12), current non-smokers including occasional smokers with less than one CPD (*n* = 3706), and current pipe or cigar smokers (*n* = 89) to restrict analysis to daily cigarette smokers. We further excluded participants with missing cotinine data due to insufficient urine volume (*n* = 66), and participants with diluted or concentrated urine samples, i.e. urinary creatinine values < 300 or > 3000 mg/L (*n* = 117). Finally, we excluded one participant with > = 100 CPD, leaving 823 current smokers for analysis.

### Urinary markers

We determined total cotinine, i.e. the sum of cotinine and cotinine-N-glucuronide, and total trans-3′-hydroxy-cotinine, i.e. the sum of trans-3′-hydroxy-cotinine and its glucuronides, in urine samples of participants provided at baseline of HNR between 2000 and 2003. These samples were frozen at − 20 °C until 2013 when total cotinine and total trans-3′-hydroxy-cotinine (hereafter referred to as cotinine or 3OH-cotinine, respectively) were measured after enzymatic hydrolyses by two-dimensional liquid chromatography linked with tandem mass spectrometry and quantification via isotope dilution, as described before [12]. We additionally calculated the sum and the ratio of 3OH-cotinine and cotinine. Urinary creatinine was measured by a contract laboratory [12] and used as marker for urinary density.

### Socioeconomic status

We primarily analysed occupational SES, using the International Socio-Economic Index of occupational status (ISEI) [14]. ISEI was constructed for men and women on the basis of age, education, and income resulting in a continuous SES score ranging from 11 for the lowest to 89 for the highest occupational status [14]. We categorised ISEI based on the distribution of the study population, separately for men and women: We combined the three middle quintiles to distinguish between the highest 20% (high), the middle 60% (intermediate), and the lowest 20% (low ISEI).

ISEI-scores referred to occupations coded by the International Standard Classification of Occupations (ISCO-08) [15]. We utilised occupational histories to select the last job that participants held at baseline examination and recoded German occupational codes into ISCO-08. Occupational histories were collected for 97% of the participants of the second follow-up of HNR, which were 52% of the current smokers at baseline in this analysis. For the remaining participants without occupational history, we coded textual information on the last job that was collected in baseline interviews into ISCO-08.

Further, we referred to the participants’ last job as white- or blue-collar occupation, by dichotomising the first digit of ISCO-08 (white-collar 1–4, blue-collar 5–9).

Educational SES was derived by categorising years of school education and vocational attainment into ≤10, 11–13, 14–17, and ≥ 18 years according to ISCED 1997 [16].

### Statistical analysis

We described the distribution of CPD and cotinine by median and interquartile range (IQR), and presented boxplots and histograms for categories of SES. The correlation of CPD and cotinine was displayed by scatterplots and included locally estimated scatterplot smoothing (loess) curve with 95% confidence interval to show potential non-linearity. We determined the appropriate functional form of CPD for cotinine-prediction by fractional polynomials including comparison of log-likelihoods [17] and applied it in subsequent analyses.

We analysed the correlation of CPD and cotinine by Pearson’s correlation coefficient ‘r’ in total and in subgroups of age (< 50, 50–59, 60–69, ≥70 years) and SES. In a sensitivity analysis for Pearson’s r, we re-included extreme creatinine values.

In addition to CPD, we investigated further potential predictors for cotinine in multiple linear regression models: age, urinary creatinine [mg/L], ISEI, body mass index (BMI), weight [kg], height [cm], daytime of body-fluid sampling (blood sampling as proxy variable for subsequent urine sampling), and finally – as potential proxy for the time of the last cigarette – the last meal or the last coffee or tea consumption before sampling, respectively. We selected further predictors by backward elimination, dropping variables with *p*-values > 0.1. Regardless of statistical significance, ISEI and age were included in the final model. Based on the final model, we displayed expected cotinine values for groups of CPD (1–5, 6–15, 16–25, 26–35, > 35) in boxplots. We explored the goodness of fit by adjusted r-squares (R^2^) of several modifications of the model. Main analyses were also performed for 3OH-cotinine.

All analyses were stratified for gender. Statistical analyses were accomplished using SAS 9.4 (SAS Institute Inc., Cary, NC, USA).

## Results

### Study population

Table 1 shows the characteristics of 437 male and 386 female smokers. In this elderly study population, men were slightly older than women. ISEI was equally distributed for men and women following the predefined categories. In contrast, women’s last occupation was more frequently a white-collar job (83%) compared to men (55%), but men spent more time in the educational and vocational system than women. The median for CPD was 20 (IQR 12–25) in men and 19 (IQR 10–20) in women, corresponding to one cigarette pack smoked daily. Men had higher median urinary concentrations of cotinine (3652 μg/L, IQR 2279–5422 μg/L) and 3OH-cotinine (6146 μg/L, IQR 3521–10,524 μg/L) than women (cotinine: 3127 μg/L, IQR 1692–4920 μg/L, 3OH-cotinine: 4815 μg/L, IQR 2418–9692 μg/L). Urinary density measured by creatinine was higher in men. Samples were collected from most participants during morning until midday.

**Table 1:** Distribution of cigarette smoker characteristics

	**Men**		**Women**	
	**n**	**%**	**n**	**%**
Total	437	53.1	386	46.9
Age [years]				
< 50	80	18.3	87	22.5
50–59	191	43.7	194	50.3
60–69	136	31.1	84	21.8
> =70	30	6.9	21	5.4
ISEI				
High (m:> 62.39–88.96)/(w:> 56.00–88.96)	85	19.5	71	18.4
Intermediate (m:> 25.95–62.39)/(w:> 27.91–56.00)	249	57.0	231	59.8
Low (m:> = 11.74–25.95)/(w:> = 11.74–27.91)	99	22.7	77	19.9
Missing	4	0.9	7	1.8
Blue−/white-collar occupation				
White collar	239	54.7	320	82.9
Blue collar	192	43.9	59	15.3
Missing	6	1.4	7	1.8
Years of education				
> =18	34	7.8	24	6.2
14–17	107	24.5	50	13.0
11–13	262	60.0	256	66.3
< =10	33	7.6	56	14.5
Missing	1	0.2	0	0.0
	**Median**	**IQR**^**a**^	**Median**	**IQR**^**a**^
Age [years]	56	51–63	54	50–60
Cigarettes per day	20	12–25	19	10–20
Cotinine [μg/L]^a^	3652	2279–5422	3127	1692–4920
3OH-cotinine [μg/L]^a^	6146	3521–10,524	4815	2418–9692
Sum of cotinine^a^ and 3OH-cotinine^a^ [μg/L]	10,093	6334–15,542	8257	4563–14,539
Ratio 3OH-cotinine^a^/cotinine^a^	1.80	1.26–2.53	1.77	1.18–2.62
Creatinine in urine [mg/L]	1280	860–1760	895	540–1315
Daytime of body fluid sampling^b^ [hours:minutes]	10:36	9:20–12:15	10:33	9:22–12:06
Body mass index [kg/m^2^]	27	25–29	26	23–29
Height [cm]	175	170–180	163	159–167
Weight [kg]	82	74–90	68	61–78
Last meal before sampling^b^ [hours]	12	4–14	12	4–14
Last coffee/tea consumption before sampling^b^ [hours]	6	4–13	5	4–14

*ISEI* International Socio-Economic Index of occupational status, *m* men, *w* women, *IQR* interquartile range, *3OH-cotinine* trans-3′-hydroxy-cotinine

Missing values for men | women: creatinine 38 | 38; daytime of sampling 2 | 4; body mass index 1 | 3; height 1 | 2; weight 1 | 3; last meal 4 | 5; last coffee or tea 155 | 150

^a^ In urine, including respective glucuronides

^b^ Blood sampling as proxy for urine sampling

### Daily cigarette consumption and urinary cotinine by SES

**Fig. 1:**
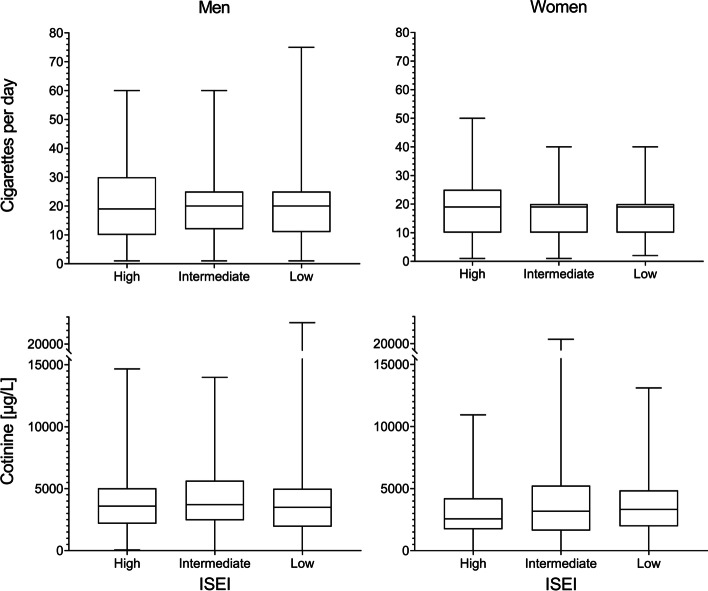
Boxplots of cigarettes per day and cotinine for categories of ISEI (International Socio-Economic Index of occupational status) for men and women

The median values of CPD were 19 and 20 in almost all subgroups of SES, which was also the case for age groups, except lower median CPD in older men and women (Table 2). Figure 1 displays the equal median CPD values for subgroups of ISEI in boxplots, with larger IQR for men and women with high ISEI. All ISEI subgroups for men and women showed peaks of self-reported numbers of daily cigarettes consumption at 10, 15, 20, 25, and 30 CPD (Fig. S1, additional file 1). Subjects with low ISEI reported less frequently 40 CPD, but more frequently 20 CPD.

**Table 2:** Cigarettes per day (CPD), urinary cotinine [μg/L], and correlations of ln (CPD) and cotinine in subgroups

	Men						Women					
	n	CPD^b^	IQR	Cotinine^b^	IQR	r^a^	n	CPD^b^	IQR	Cotinine^b^	IQR	r^a^
Total	437	20	12–25	3652	2279–5422	0.40	386	19	10–20	3127	1692–4920	0.40
Age [years]												
< 50	80	20	15–25	4140	2778–5219	0.43	87	20	15–22	3497	1660–5564	0.37
50–59	191	20	15–30	4329	2699–6112	0.31	194	19	10–20	3086	1776–5170	0.38
60–69	136	19	10–25	3186	1802–4674	0.42	84	15	9–20	3082	1701–4512	0.39
> =70	30	11	6–20	2155	1418–4078	0.42	21	10	5–15	1867	633–2544	0.62
ISEI												
High	85	19	10–30	3593	2199–5035	0.48	71	19	10–25	2566	1727–4221	0.47
Intermediate	249	20	12–25	3716	2458–5652	0.39	231	19	10–20	3177	1615–5249	0.43
Low	99	20	11–25	3493	1925–4992	0.35	77	19	10–20	3323	1987–4701	0.28
Missing	4	18	14–25	3895	1844–5640		7	20	18–20	3683	2553–4789	
Blue−/white-collar occupation												
White collar	239	20	12–30	3793	2315–5520	0.41	320	19	10–20	2927	1666–4840	0.41
Blue collar	192	20	12–25	3489	2180–5191	0.39	59	19	12–25	3542	1987–5617	0.37
Missing	6	18	15–20	3895	2998–7146		7	20	18–20	3683	2553–4789	
Education [years]												
> =18	34	20	10–30	3772	2159–5210	0.44	24	20	9–20	1995	1136–2672	0.45
14–17	107	20	15–25	3736	2390–6345	0.43	50	17	10–20	2702	1692–4182	0.42
11–13	262	20	12–25	3654	2198–5227	0.40	256	19	10–20	3155	1713–5084	0.41
< =10	33	20	15–30	3493	2283–5424	0.19	56	19	11–20	3760	2445–5493	0.37
Missing	1	30	30–30	6904	6904–6904		0					

*IQR* interquartile range, *ISEI* International Socio-Economic Index of occupational status

^a^ Pearson correlation coefficient for ln (CPD) and cotinine

^b^ Median

Median cotinine was similar in subgroups of SES, with a slight increase with higher education and in white-collar jobs in men (Table 2). In women, median cotinine increased with lower SES (e.g. median (IQR) high ISEI 2566 (1727–4221) μg/L, low ISEI 3323 (1987–4701) μg/L) (Fig. 1). Median cotinine was lower in the oldest age group > = 70 years. The patterns of median 3OH-cotinine according to SES and age were similar to those of cotinine (Table S1, additional file 1). In scatter plots of cotinine and continuous ISEI, no correlation appeared in men as indicated by the linear regression line, whereas cotinine slightly decreased with increasing ISEI in women (Fig. S2, additional file 1).

### Association of cigarettes per day and cotinine

**Fig. 2:**
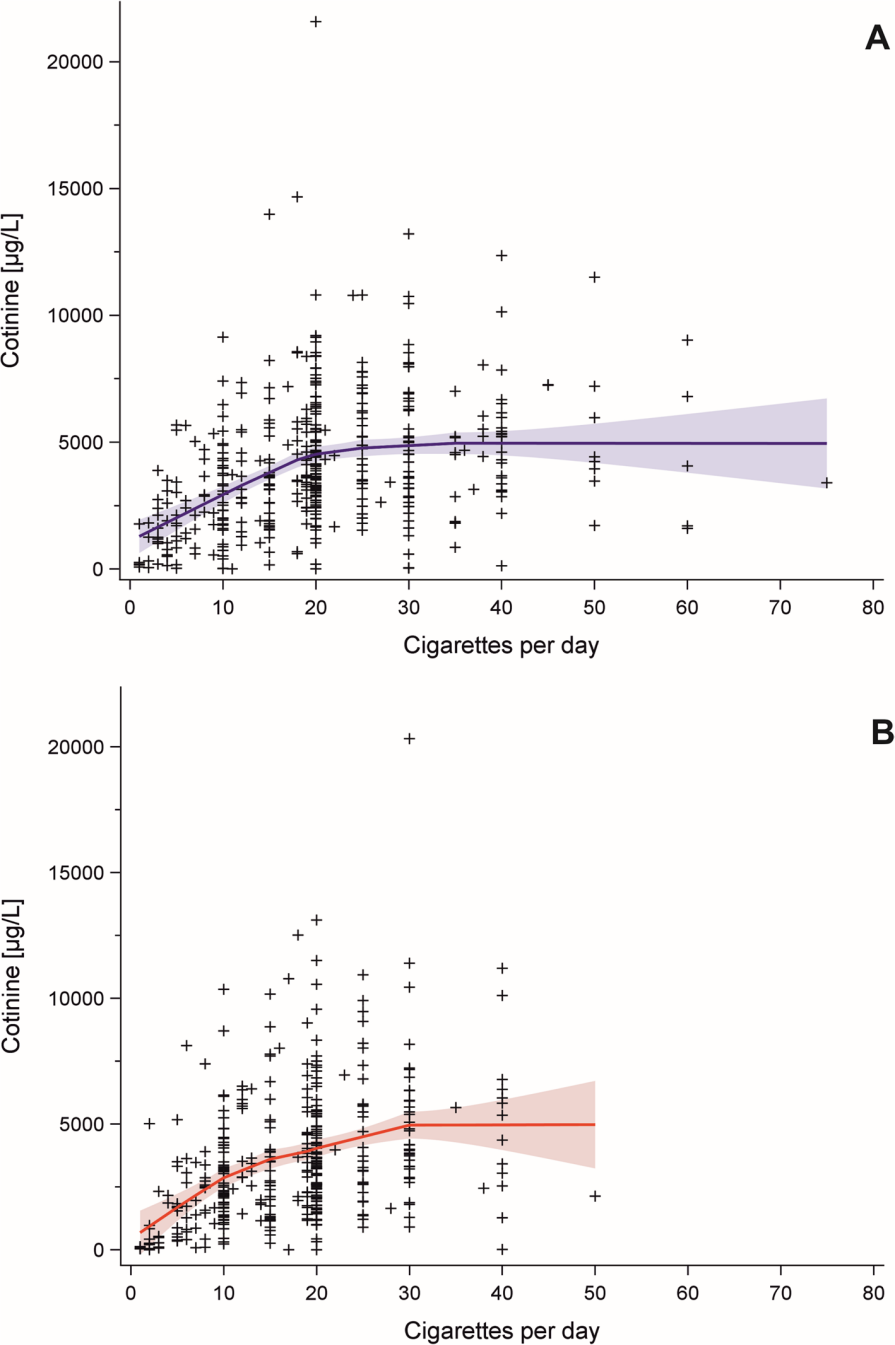
Cotinine and cigarettes per day including loess fit curve with 95% confidence interval in (**A**) men (smoothing parameter 0.76) and (**B**) women (smoothing parameter 0.63)

Figure 2 shows the distribution of CPD and cotinine for men and women. The loess curves levelled off at 20 CPD, more clearly in men. The corresponding loess curves for 3OH-cotinine were comparable to cotinine, with a less pronounced flattening (Fig. S3, additional file 1). We determined the natural logarithm of CPD as functional form of CPD to predict cotinine in men and women by goodness of fit. This form of CPD also corresponded to the graphical analyses by loess curves.

We found a moderate correlation of CPD and urinary cotinine, as Pearson’s r was 0.4 for both, men and women (Table 2). Whereas correlations in subgroups of age did not indicate differences in terms of trends, correlations increased with higher SES. For example, Pearson’s r for low, intermediate, and high ISEI was 0.35, 0.39, and 0.48 in men, and 0.28, 0.43, and 0.47 in women. Correlations with CPD were slightly lower for 3OH-cotinine (men 0.34, women 0.32) (Table S1) and the sum of cotinine and 3OH-cotinine (men 0.39, women 0.37). This was also found for cotinine when we included urines with extreme creatinine values (Pearson’s r men 0.39, women 0.33).

In addition to CPD, urinary creatinine significantly improved the goodness of fit of the multiple linear regression model in men as well as women. Using backward elimination, further remaining variables were age, ISEI (only women), and weight (only men). Table 3 presents the parameters of the final model including CPD, creatinine, age and ISEI together with adjusted R^2^ (0.33 in men and women). Again, results for 3OH-cotinine were similar to cotinine (adjusted R^2^ men 0.31, women 0.35) (Table S2, additional file 1). Cotinine values predicted by the final model are presented in boxplots for categories of CPD (Fig. S4, additional file 1). Starting with median expected cotinine of 1700 μg/L in male and 900 μg/L in female low-dose smokers (1–5 CPD), it was near 4000 μg/L for smokers of about one daily cigarette pack (16–25 CPD), and did marginally exceed 5000 μg/L at higher CPD consumption in men.

**Table 3:** Estimated parameters of multiple linear regression for potential predictors of urinary cotinine [μg/L] in current smokers

Men (***n*** = 395^a^)	$\hat{\boldsymbol{\upbeta}}$	95% LCL^b^	95% UCL^b^	Adjusted R^**2**^
Intercept	1180.32	− 954.12	3314.75	0.333
Ln (cigarettes per day)	1318.20	1028.34	1608.06	
Age [years/10]	− 472.02	− 762.03	−182.00	
Creatinine in urine [mg/L]	1.54	1.20	1.87	
ISEI^c^	−59.12	− 171.30	53.05	
**Women (*n*** **= 341**^a^**)**				
Intercept	506.29	− 1879.78	2892.37	0.333
Ln (cigarettes per day)	1378.95	1047.38	1710.52	
Age [years/10]	− 306.34	− 639.12	26.44	
Creatinine in urine [mg/L]	1.76	1.37	2.16	
ISEI^c^	−176.81	−300.91	−52.71	

^a^ 42 men and 45 women excluded because of missing values

^b^ Lower and upper confidence limit

^c^ International Socio-Economic Index of occupational status, continuous variable with range divided by 10: 1 (low) to 9 (high)

When exploring changes of model fit in further models (Table S3, additional file 1), we found considerable improvement of the model fit only by additional adjustment for the ratio of cotinine/3OH-cotinine (adjusted R^2^ men 0.40, women 0.41). Replacing ISEI with years of education produced the same results, except a switch to a marginally positive association in men.

## Discussion

In this study, we analysed the associations of CPD, urinary cotinine, and SES in an elderly German population of current smokers. Most men and women reported to smoke one pack of cigarettes per day (20 CPD). While the reported median smoking intensity did not vary by SES, CPD reports were more variable in men and women with high SES. The association of CPD and cotinine levelled off at about 20 CPD, and the logarithm of CPD was the best functional form to predict cotinine. The correlation between CPD and cotinine increased with high SES, indicating a higher precision of self-reports. In regression models, CPD and creatinine were the main predictors of cotinine. In women, cotinine slightly decreased with higher SES, as confirmed in the multiple regression. All findings for cotinine were similar for 3OH-cotinine.

For our study we utilised data of a population-based cohort with largely available information on smoking and occupational and educational SES, and we were able to apply highly sensitive urinary markers for smoking.

Although self-reported average daily cigarette consumption is the common measure of smoking intensity in health studies, a more precise measure of current smoking exposure would have included the exact number of cigarettes in the days before examination, and/or the time of the last cigarette. We used the last meal and the last consumption of coffee or tea before sampling as a proxy for the latter, but did not find associations with cotinine. However, average and recent CPD were found to be highly correlated [6]. We also did not have information on individual puffing behaviour, which was found to be a mediator for the association of CPD and salivary cotinine [18], and thus could have contributed to a better predictive model for urinary cotinine. We also lacked data on the magnitude of nicotine dependence beyond CPD. In particular, the time to the first cigarette after awakening was found to be an additional predictor for cotinine [19, 20]. We did not consider cigarette brands, which may vary by nicotine content. However, it is not clear to which extent different nicotine contents are reflected in cotinine concentrations, as they might be compensated by inhalation behaviour [21].

We observed a well-known, wide variation of cotinine concentrations at each level of CPD which partially can be attributed to genetic differences in nicotine metabolism [4, 22]. The nicotine metabolism rate is commonly displayed by the ratio of 3OH-cotinine/cotinine, additionally by the ratio of cotinine glucuronide/cotinine [23]. Our additional adjustment for the ratio of 3OH-cotinine/cotinine increased the model fit. However, urinary nicotine metabolite ratios varied in previous studies, probably dependent on the type of measurement [24]. Differences in nicotine metabolism also likely account for the reduced cotinine values in women [4]. The sum of cotinine and 3OH-cotinine might more comprehensively reflect nicotine uptake than single metabolites [25], but we found a slightly higher correlation with CPD for cotinine. As mentioned, variation of nicotine metabolites might be reduced by additional information on puffing behaviour, nicotine dependence, and more detailed recent cigarette exposure.

Further, retrospective self-reports of CPD tend to show a digit preference, i.e. reports of multiples of 10 or 5, which increase at higher CPD [26] and were also apparent in our data. More frequent reports of one daily pack and decreased correlation of CPD and cotinine indicated lower precision of CPD reports for lower SES [5].

The flattening we observed in the association between CPD and cotinine confirms results of several studies, regardless of the body fluid (plasma, saliva, or urine) that was used to determine cotinine [6–8, 18, 27]. Investigating non-linearity, we selected the logarithm of CPD to appropriately depict this association, while most other studies remained with visual evidence of the plateau effect. Some studies found improved model fits with an additional quadratic term of CPD [5, 27, 28], or setting a cut-off at 20 CPD in regression analyses [18, 27].

In our multiple regression model, urinary creatinine was confirmed to be an important predictor for cotinine [28]. In contrast to our results for CPD, creatinine, and to results of some other studies, we found only weak effects for BMI/weight [29], and also age, the latter a possible consequence of the narrow age range in our study population. A gender effect was not investigated here, as all models were stratified by gender.

We found a slightly negative association of occupational and educational SES and cotinine in women, but no obvious association in men. A negative association of SES and cotinine was observed in other studies, though varying by SES indicator: Higher cotinine concentrations were found for lower education, but not income or occupation in the FINRISK study [10], and deprivation, but not lower occupation in an English survey [11]. A Czech study on CPD and thiocyanate, another biomarker for smoking, showed higher levels for low education [30]. Results in these studies were adjusted for CPD, however, as linear variable. Further, an income/wealth-based SES index was negatively associated with cotinine when adjusting for nicotine dependence (including CPD), but only in the subgroup of unemployed subjects [31].

Different causes for the widely observed flattening of cotinine values at higher CPD may be considered:

First, there is possibly a biological maximum for the uptake and metabolism of nicotine. A biological saturation was also discussed to explain flattening cancer risks in heavy smokers [9, 32]. However, increasing lung cancer risks were also reported at higher cotinine concentrations [33, 34], and specific associations of other (carcinogenic) tobacco smoke constituents with CPD were observed [8]. Thus, it seems problematic to infer a biological saturation for cotinine from the association of CPD and cancer.

Another possible cause is compensatory smoking behaviour, i.e. less inhalation in heavy smokers. Similar associations of CPD and cotinine including a flattening were shown for smokers of cigarettes with regular compared to reduced nicotine content [27]. Most studies on compensation investigated effects of nicotine reduced cigarettes, but not separately for light/heavy smokers, and with different results [35–37]. In a comparison of large surveys with over two decades in between, cotinine concentrations remained constant while CPD substantially decreased, which was attributed to compensatory smoking [38].

Finally, information bias of self-reported CPD could lead to flattening CPD-cotinine-curves, assuming an underreporting of CPD in the middle CPD section with corresponding high cotinine values. An independent effect of SES on cotinine would have indicated information bias, whereas in particular biological saturation should not differ by SES. Stronger compensation could also be associated with lower SES due to higher nicotine dependence [10, 39] or financial reasons [30, 40]. I.e., being financially restricted could increase pressure to satisfy nicotine needs with a relatively lower number of cigarettes per day, with 20 CPD being more frequently reported for low SES in our study. However, we found only a weak association between SES and cotinine either with or without adjustment for CPD.

## Conclusions

Self-reported cigarette smoking intensity was similar across SES groups. Our study results suggest a slight decrease for precision of reports with lower SES in men and women. Urinary cotinine concentrations were slightly dependent on SES in women. In summary, we did not find strong evidence that self-reports on smoking intensity were biased by SES. This is in line with our previous analysis on self-reported current smoking status and SES. Thus, for similar study populations and analyses of SES and health, our results do not raise strong concerns about varying validity of self-reported smoking.

## Supplementary Information

**Additional file 1 Table S1.** Cigarettes per day (CPD), urinary trans-3‘-hydroxy-cotinine (3OH-cotinine) [μg/L], and correlations of ln (CPD) and 3-OH-cotinine in subgroups. **Table S2.** Estimated parameters of a multiple linear regression model for potential predictors of urinary trans-3‘-hydroxy-cotinine [μg/L] in current smokers. **Table S3.** Variations of the multiple linear regression model for potential predictors^a^ of urinary cotinine [μg/L] in current smokers. **Figure S1.** Histograms of cigarettes per day including means and standard deviations (std) by International Socio-Economic Index of occupational status (ISEI) and gender. **Figure S2.** Cotinine and ISEI (International Socio-Economic Index of occupational status) including linear regression line for men (**A**) and women (**B**). **Figure S3.** Trans-3’-hydroxy-cotinine and cigarettes per day including loess fit curve with 95% confidence interval in (**A**) men (smoothing parameter 0.94) and (**B**) women (smoothing parameter 0.86). Fig. S4. Boxplots for expected values of urinary cotinine (predicted by ln (cigarettes per day), creatinine, age, and International Socio-Economic Index of occupational status) for categories of cigarettes per day, in men and women. Outliers with > 1.5 interquartile range (IQR) distance from IQR bounds displayed separately.

## Data Availability

Data from the Heinz-Nixdorf Recall study are not publicly available, but can be obtained by filling a request for data use and explaining the aim of the planned analyses (https://imibe.uk-essen.de/).

## Abbreviations

3OH: Trans-3′-hydroxy
CPD: Cigarettes per day
HNR: Heinz-Nixdorf Recall Study
IQR: Interquartile range
ISCO: International Standard Classification of Occupations
ISEI: International Socio-Economic Index of occupational status
R^2^: R-square
SES: Socioeconomic status
