# Supplementary material for: Smoking intensity and urinary nicotine metabolites by socioeconomic status in the Heinz Nixdorf Recall study
Source: BMC Public Health. 2022 Feb 14;22:302. doi: 10.1186/s12889-022-12609-y (PMC8842804; doi:10.1186/s12889-022-12609-y)
Supplement: Supplementary file 1 — Additional file 1 Table S1. Cigarettes per day (CPD), urinary trans-3‘-hydroxy-cotinine (3OH-cotinine) [μg/L], and correlations of ln (CPD) and 3-OH-cotinine in subgroups. Table S2. Estimated parameters of a multiple linear regression model for potential predictors of urinary trans-3‘-hydroxy-cotinine [μg/L] in current smokers. Table S3. Variations of the multiple linear regression model for potential predictorsa of urinary cotinine [μg/L] in current smokers. Figure S1. Histograms of cigarettes per day including means and standard deviations (std) by International Socio-Economic Index of occupational status (ISEI) and gender. Figure S2. Cotinine and ISEI (International Socio-Economic Index of occupational status) including linear regression line for men (A) and women (B). Figure S3. Trans-3’-hydroxy-cotinine and cigarettes per day including loess fit curve with 95% confidence interval in (A) men (smoothing parameter 0.94) and (B) women (smoothing parameter 0.86). Fig. S4. Boxplots for expected values of urinary cotinine (predicted by ln (cigarettes per day), creatinine, age, and International Socio-Economic Index of occupational status) for categories of cigarettes per day, in men and women. Outliers with > 1.5 interquartile range (IQR) distance from IQR bounds displayed separately. [file 12889_2022_12609_MOESM1_ESM.pdf]

SUPPLEMENTARY MATERIAL

Table S1. Cigarettes per day (CPD), urinary trans-3'-hydroxy-cotinine (3OH-cotinine) [µg/L], and correlations of ln(CPD) and 3-OH-cotinine in subgroups

	Men						Women					
	n	CPD[b]	IQR	3OH-cotinine[b]	IQR	r[a]	n	CPD[b]	IQR	3OH-cotinine[b]	IQR	r[a]
Total	437	20	12-25	6146	3521-10,524	0.34	386	19	10-20	4815	2418-9692	0.32
Age group												
<50	80	20	15-25	7372	4596-10,871	0.42	87	20	15-22	4874	2265-9451	0.34
50-59	191	20	15-30	7184	3921-11,898	0.25	194	19	10-20	4815	2722-9976	0.26
60-69	136	19	10-25	5071	2861-8482	0.37	84	15	9-20	5743	2418-10,041	0.37
>=70	30	11	6-20	4059	2160-7585	0.41	21	10	5-15	2608	1106-4170	0.51
ISEI												
High	85	19	10-30	5939	3593-10,854	0.49	71	19	10-25	4950	2359-8950	0.38
Intermediate	249	20	12-25	6346	3644-10,376	0.30	231	19	10-20	4713	2509-9802	0.36
Low	99	20	11-25	5765	2826-9619	0.32	77	19	10-20	4874	2053-9825	0.16
Missing	4	18	14-25	9026	4235-10,606		7	20	18-20	4494	3979-11,459	
Blue-/white-collar												
White collar	239	20	12-30	6819	3666-10,834	0.37	320	19	10-20	4707	2374-9437	0.33
Blue collar	192	20	12-25	5583	3314-9370	0.33	59	19	12-25	5869	2788-10,497	0.24
Missing	6	18	15-20	9026	3644-11,554		7	20	18-20	4494	3979-11,459	
Education												
>=18 years	34	20	10-30	7758	4080-12,267	0.40	24	20	9-20	2363	1636-6906	0.47
14–17 years	107	20	15-25	6334	3567-11,304	0.39	50	17	10-20	4841	2258-8647	0.36
11–13 years	262	20	12-25	6134	3418-10,001	0.33	256	19	10-20	4707	2628-9455	0.33
<=10 years	33	20	15-30	4996	3719-7874	0.29	56	19	11-20	6808	2510-11,825	0.23
Missing	1						0					

IQR – interquartile range, CI – confidence interval, ISEI – International Socio-Economic Index of occupational status

[a] Pearson correlation coefficient for ln(CPD) and trans-3'-hydroxy-cotinine

[b] Median

Table S2. Estimated parameters of a multiple linear regression model for potential predictors of urinary trans-3'-hydroxy-cotinine [µg/L] in current smokers

**Men (n=395[a])**	$\hat{\beta}$	**95% LCL[b]**	**95% UCL[b]**	**adjusted R^2**
Intercept	-1401.54	-6322.51	3519.42	0.309
Ln(cigarettes per day)	2586.03	1917.75	3254.31	
Age [years/10]	-660.77	-1329.41	7.86	
Creatinine in urine [mg/L]	3.86	3.09	4.63	
ISEI[c]	34.17	-224.46	292.79	
**Women (n=341[a])**				
Intercept	-1820.41	-7228.33	3587.51	0.350
Ln(cigarettes per day)	2625.94	1874.46	3377.43	
Age [years/10]	-565.11	-1319.34	189.11	
Creatinine in urine [mg/L]	5.03	4.14	5.93	
ISEI[c]	-125.13	-406.40	156.13	

[a] 42 men and 45 women excluded because of missing values

[b] Lower and upper confidence limit

[c] International Socio-Economic Index of occupational status, continuous variable with range divided by 10: 1 (low) to 9 (high)

Table S3. Variations of the multiple linear regression model for potential predictors[a] of urinary cotinine [µg/L] in current smokers

	Men				Women			
	$\widehat{\beta}$	95% LCL	95% UCL	Adjusted R^2	$\widehat{\beta}$	95% LCL	95% UCL	Adjusted R^2
Including weight				0.339				0.335
Including body mass index				0.335				0.334
Including ratio 3OH-cotinine/cotinine				0.396				0.415
Removing creatinine				0.183				0.168
Standardising cotinine by creatinine (instead adjustment)				0.167				0.212
Replacing cotinine with ln(cotinine)				0.280				0.348
Replacing ISEI with education				0.333				0.333
Intercept	484.78	-2154.97	3124.52		1423.28	-1372.97	4219.53	
ln(Cigarettes per day)	1313.90	1022.79	1605.01		1386.21	1056.90	1715.51	
Creatinine [mg/L]	1.53	1.19	1.86		1.72	1.33	2.12	
Age [years/10]	-47.97	-77.00	-18.95		-278.23	-606.79	50.34	
Education [years]	35.87	-63.57	135.31		-135.04	-238.33	-31.74	

LCL – lower confidence limit, UCL – upper confidence limit, 3OH-cotinine – trans-3‘-hydroxy-cotinine, ISEI – International Socio-Economic Index of occupational status

[a] Original model: ln(cigarettes per day), creatinine, age, ISEI

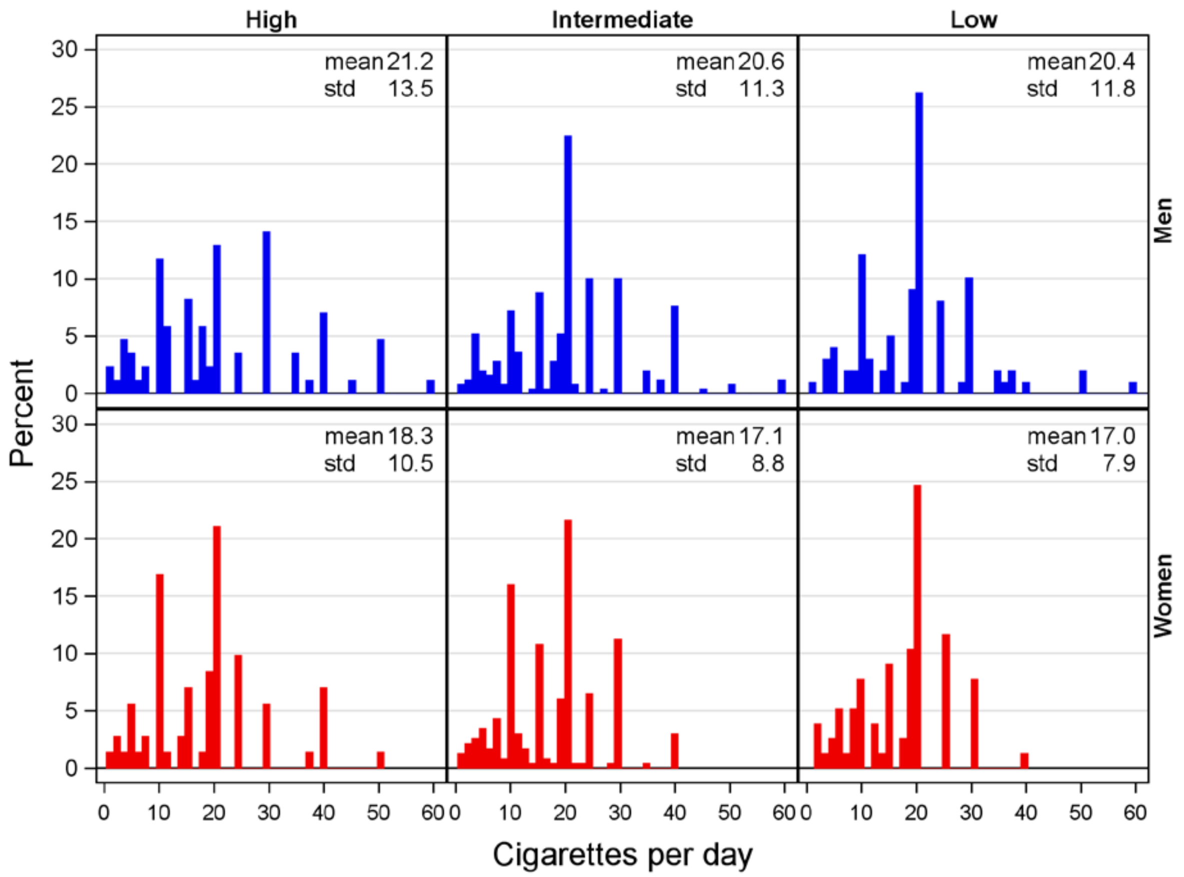

Figure S1. Histograms of cigarettes per day including means and standard deviations (std) by International Socio-Economic Index of occupational status (ISEI) and gender

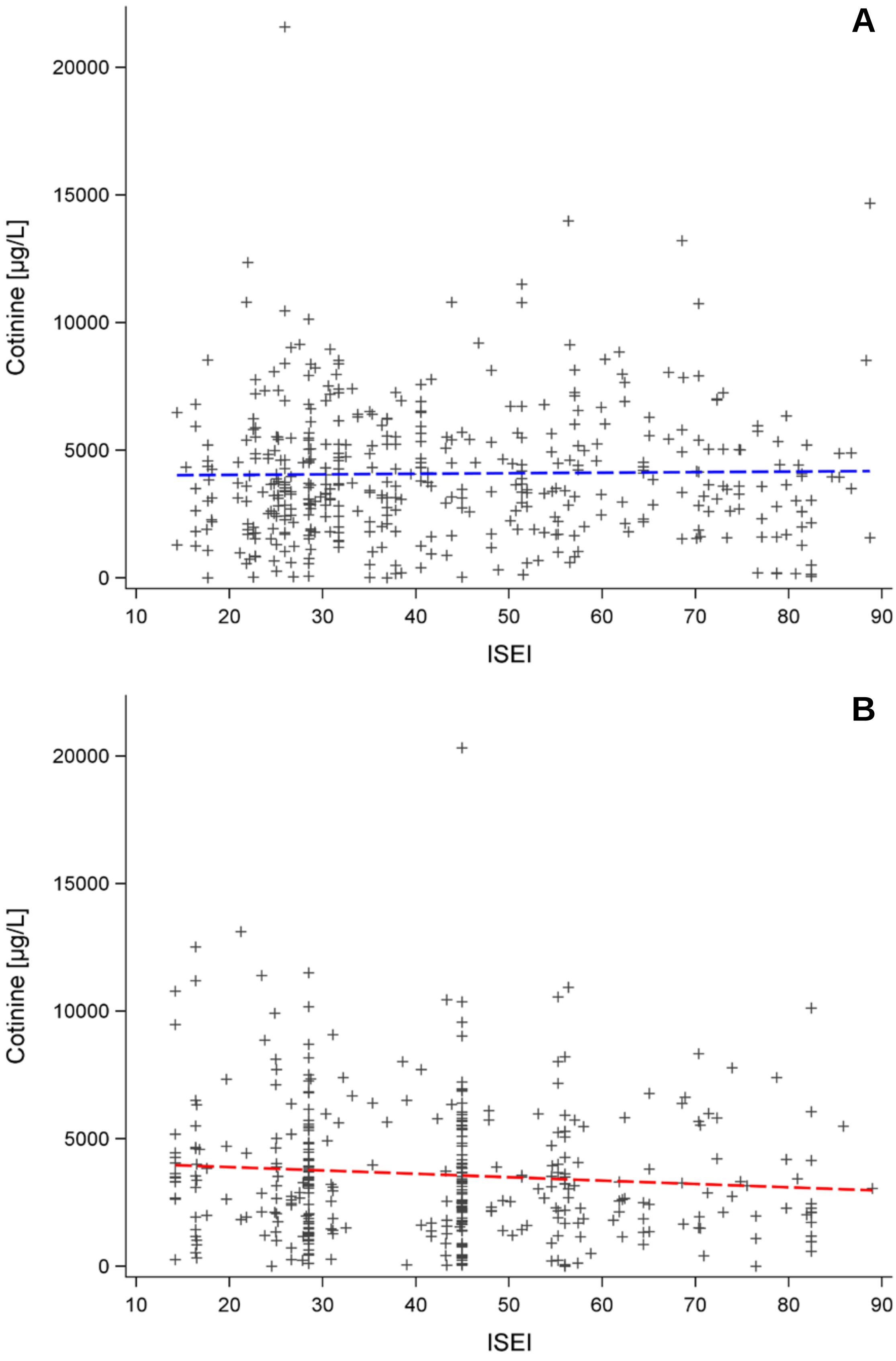

Figure S2. Cotinine and ISEI (International Socio-Economic Index of occupational status) including linear regression line for men (**A**) and women (**B**)

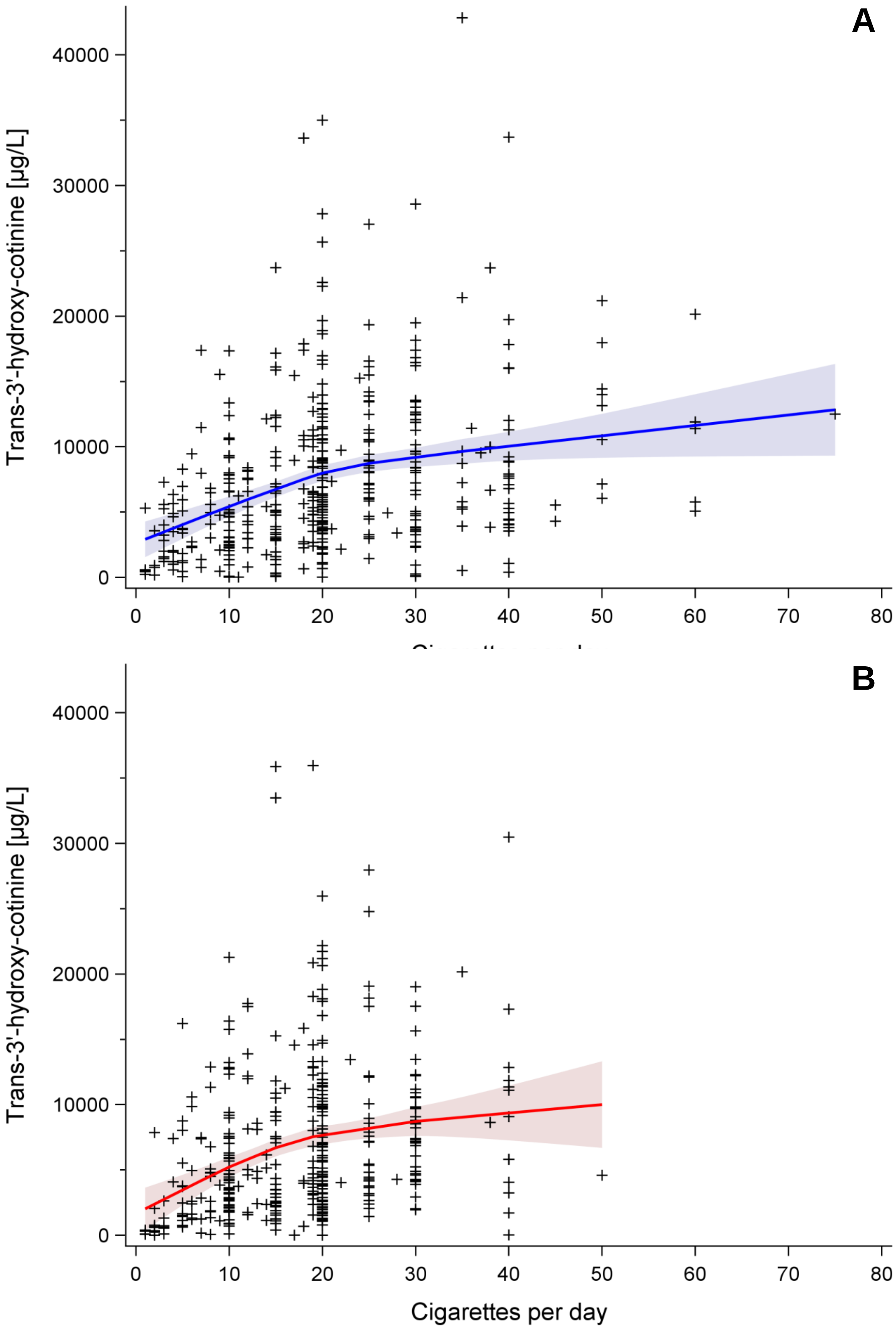

Figure S3. Trans-3'-hydroxy-cotinine and cigarettes per day including loess fit curve with 95% confidence interval in (**A**) men (smoothing parameter 0.94) and (**B**) women (smoothing parameter 0.86)

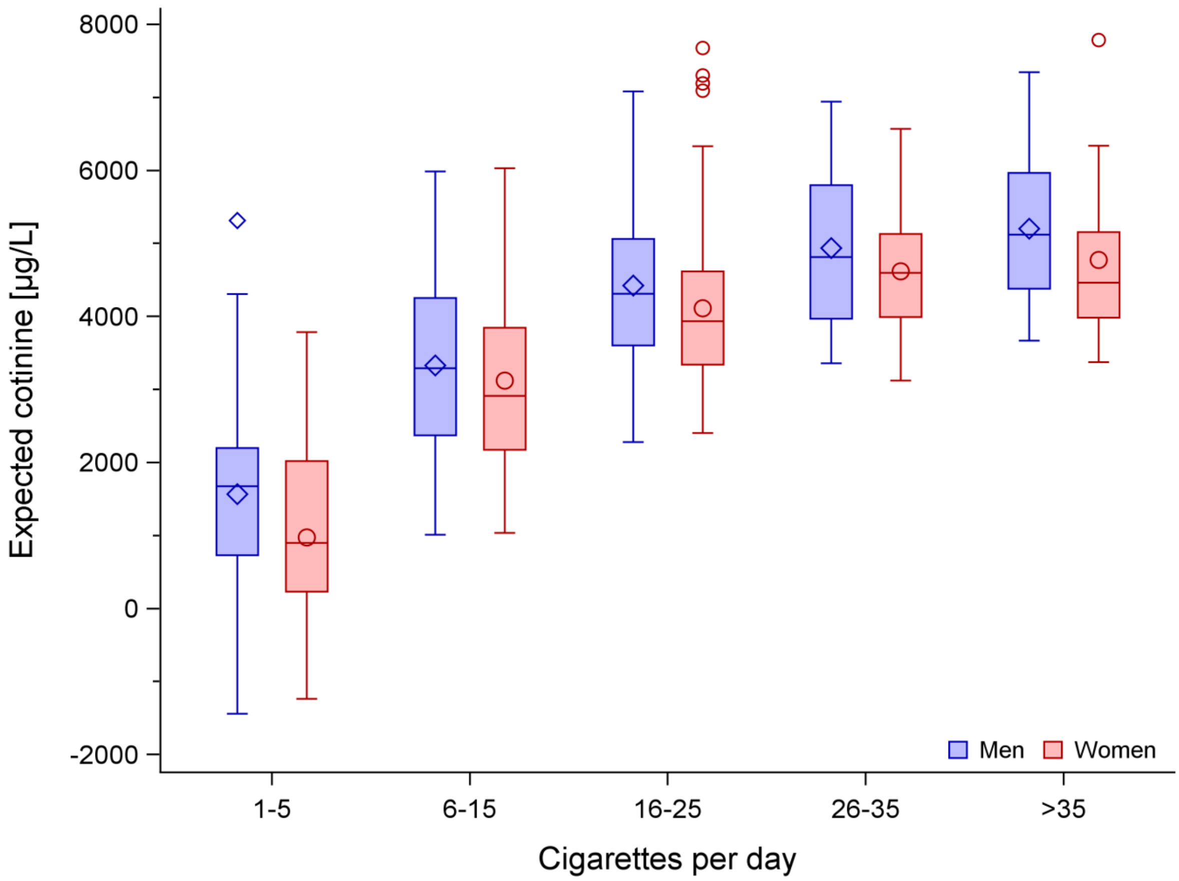

Figure S4. Boxplots for expected values of urinary cotinine (predicted by ln(cigarettes per day), creatinine, age, and International Socio-Economic Index of occupational status) for categories of cigarettes per day, in men and women. Outliers with >1.5 interquartile range (IQR) distance from IQR bounds displayed separately
